# Identification of functional networks in resting state fMRI data using adaptive sparse representation and affinity propagation clustering

**DOI:** 10.3389/fnins.2015.00383

**Published:** 2015-10-16

**Authors:** Xuan Li, Haixian Wang

**Affiliations:** Key Lab of Child Development and Learning Science of Ministry of Education, Institute of Child Development and Education, Research Center for Learning Science, Southeast UniversityNanjing, China

**Keywords:** adaptive sparse representation, affinity propagation, functional connectivity, association matrix, resting-state fMRI

## Abstract

Human brain functional system has been viewed as a complex network. To accurately characterize this brain network, it is important to estimate the functional connectivity between separate brain regions (i.e., association matrix). One common approach to evaluating the connectivity is the pairwise Pearson correlation. However, this bivariate method completely ignores the influence of other regions when computing the pairwise association. Another intractable issue existed in many approaches to further analyzing the network structure is the requirement of applying a threshold to the association matrix. To address these issues, we develop a novel scheme to investigate the brain functional networks. Specifically, we first establish a global functional connection network by using the Adaptive Sparse Representation (ASR), adaptively integrating the sparsity of ℓ_1_-norm and the grouping effect of ℓ_2_-norm for linear representation and then identify connectivity patterns with Affinity Propagation (AP) clustering algorithm. Results on both simulated and real data indicate that the proposed scheme is superior to the Pearson correlation in connectivity quality and clustering quality. Our findings suggest that the proposed scheme is an accurate and useful technique to delineate functional network structure for functionally parsimonious and correlated fMRI data with a large number of brain regions.

## 1. Introduction

Recently, it has been widely accepted that brain functional system is a complex network due to the features such as small-worldness, highly connected hubs and modularity (Watts and Strogatz, 1998; Bullmore and Sporns, 2009). Functional magnetic resonance imaging (fMRI), as a useful technique in the brain mapping realm, provides valuable data resource for investigating human neural functional network architecture. Even resting-state fMRI data, acquired when participants are in rest without performing any particular task, can provide meaningful information. Analyzing fMRI data from the viewpoint of network has been carried out in many studies that investigate various problems such as gender (Tian et al., 2011), intelligence (van den Heuvel et al., 2009; Song et al., 2014; Vakhtin et al., 2014), age (Meunier et al., 2009; Wang et al., 2010), memory (Ginestet and Simmons, 2011; Cao et al., 2014), and neuropsychiatric disorders such as schizophrenia (Bassett et al., 2008; van den Heuvel and Fornito, 2014) and Alzheimer's disease (Supekar et al., 2008; Zhao et al., 2012; Liu et al., 2014). Generally, the scheme first constructs an association matrix (i.e., the global functional connection network), decomposes it into sub-networks, and possibly extracts some connection-based features, such as network measures, for further analysis. Therefore, a core issue is to correctly model the functional network, which is the basis for functional brain analysis.

The functional connectivity is defined as the temporal dependency between spatially separated brain regions (Friston et al., 1993), and is conveniently represented by the association matrix when the direction of connections is not of concern. Correlation-based methods, such as pairwise Pearson correlation and partial correlation, are largely used to calculate the functional connectivity. These correlation-based methods usually achieve encouraging performance in network modeling, which may suggest that important information lies in variance as mentioned in Smith et al. (2011). The Pearson correlation is especially popular to compute the functional connectivity for its efficiency. However, one major limitation of the Pearson correlation method is that it computes the pairwise association between nodes without considering the contribution of other nodes. It may happen that some weak connections in terms of the Pearson correlation take effect if they work collectively. They constitute an intrinsic part of the brain network. Moreover, the pairwise analysis is likely to produce spuriously high values of correlation in the situation that they are actually related with multiple responded regions. The presence of the large number of faked connections could lead to over-fitting when decoding fMRI data (Liu et al., 2009).

Given the association matrix, one succedent analysis is to identify the intrinsic sub-networks by applying a threshold to the entries of the association matrix. By removing the relatively small values from the association matrix, we expect to reveal regions that have some underlying common function. It is then convenient to calculate network measures such as small-worldness, clustering coefficient, and path length, etc… (Bullmore and Sporns, 2009). However, there is not a generally agreed criterion to select an appropriate threshold, which is critical to correctly reflecting the network structure. Besides, one single value of threshold may not be suitable for the whole brain. In other words, the threshold method is not adaptive. Alternative ways of identifying sub-networks have been developed in literature. The representative methods include clustering approaches like InfoMap (Rosvall and Bergstrom, 2008; Power et al., 2011) and Normalized Cuts (NCuts) (van den Heuvel et al., 2008), and matrix factorization approaches like Independent Component Analysis (ICA) (Beckmann et al., 2005) and Principal Component Analysis (PCA) (Friston, 1998). Some of these approaches are applied either directly to fMRI time series or to similarity measures of fMRI series without taking advantage of functional connectivity information, and some still could not circumvent the problem of threshold setting.

We thus seek a novel scheme to overcome the limitations lying in the construction of the association matrix and the identification of the intrinsic sub-networks (connectivity patterns) for fMRI data. Firstly, the Adaptive Sparse Representation (ASR) (Grave et al., 2011; Lu et al., 2013; Wang et al., 2014) is introduced to construct the association matrix. In contrast with the pairwise Pearson correlation, the ASR simultaneously considers the linear relationship of one certain node with all the other nodes. It is well-known that the technique of sparse representation has been extensively used in the domain of image processing (Wright et al., 2009). Recently, the sparse representation has drawn increasing attention in the context of brain imaging and decoding (Ganesh et al., 2008; Li et al., 2009, 2014), which is beneficial to model the topological efficiency of the brain network and meanwhile lower the connection cost (Bullmore and Sporns, 2012). A few researches have studied the sparse connectivity (Haufe et al., 2010; Ryali et al., 2012) and some provide valuable information in the aspect of neurological diseases (Zhao et al., 2012; Lee et al., 2013; Wee et al., 2014). The sparsity characteristic of brain activities has been supported by some neurophysiological findings (Olshausen and Field, 1996; Quiroga et al., 2005, 2008), which are the basis for applying sparse representation-based method for neural imaging data. These findings suggest that information is encoded by a sparse set of neurons that response to a specific input stimulus (Lee et al., 2011).

To pursue a sparse solution for the sparse representation of high fMRI data, the ℓ_1_-norm regularization (Tibshirani, 1996), also known as LASSO, is a common choice in related studies (Ganesh et al., 2008; Li et al., 2009). Although the ℓ_1_-norm provides great sparsity in revealing significant connections in a functional network, it has poor stability. That is, given correlated variables, the resulted variables with the ℓ_1_-norm solution may be randomly selected (Grave et al., 2011). However, fMRI data is usually in such a case where spatially adjacent regions are likely to be highly correlated. Consequently, the utilization of the ℓ_1_-norm regularization in fMRI data deserves to be deeply studied. In the context of statistics, some remedies have been proposed to address this problem. Specifically, by combining the ℓ_1_-norm with the ℓ_2_-norm which has grouping effect on correlated data, the elastic net (Zou and Hastie, 2005) and group LASSO (Yuan and Lin, 2006) have been developed. However, the elastic net involves two tuning parameters and the group LASSO needs prior grouping information. Recently, a trace norm that seamlessly interpolates the ℓ_1_-norm and the ℓ_2_-norm, called trace LASSO, has been newly established as an ideal regularizer (Grave et al., 2011). Depending on the data at hand, the trace LASSO regularization achieves a balance between the sparsity provided by the ℓ_1_-norm and the grouping effect by the ℓ_2_-norm adaptively with only one regularization parameter. The ASR uses the trace LASSO regularizer in the linear representation, and has demonstrated good performance in subspace segmentation (Lu et al., 2013) and face recognition (Wang et al., 2014). Considering the highly correlated fMRI data of spatially adjacent brain regions, we are thus motivated to use the ASR to establish the global functional connection network in our study. Note that the anatomical connectivity of the macaque (Felleman and Van Essen, 1991; Markov et al., 2012) suggests that the connectivities between different brain areas of the macaque are highly dense. The point is that there are non-zero (albeit) weak connections among many pairs of regions. Besides, the modular and rich-club-like network architecture of the human brain remain valid (Park and Friston, 2013). This may imply that the human brain is heavily connected within the same sub-networks while sparsely connected between different sub-networks. Accordingly, we employ the trace LASSO to discover collective correlations among many regions rather than paired correlations. Unlike the Pearson correlation, the trace LASSO takes weak connections into account if they jointly contribute with others. In fact, the global linear representation modeled via the trace LASSO could be viewed as a generalization of the Pearson correlation in the sense that the Pearson correlation coefficient is the linear representation coefficient between two variables according to the regression theory. Besides, different from the ℓ_1_-norm, the trace LASSO does not pursue the sparsity greedily. Rather, it has the adaptive property and results in structured (modularity) correlation.

Secondly, we apply the Affinity Propagation (AP) clustering algorithm (Frey and Dueck, 2007) to the obtained association matrix. As a result, we identify the intrinsic network structure by clustering all nodes into non-overlapping sub-networks, avoiding the problem of threshold setting mentioned earlier. The AP clustering algorithm directly operates on the association matrix of fMRI data and takes the connection strength as a measure of similarity. More importantly, the number of clusters of AP is not required to be predetermined and can be controlled by adjusting the value of preference. The AP clustering algorithm has been used in several researches to identify brain networks in voxel-wise analysis for fMRI data, where the measure of similarity is defined by using the Euclidean distance (Zhang et al., 2011) or the Pearson correlation (Li et al., 2010). In this study, we use the ASR coefficients as the measure of similarity between regions of interest (ROIs) in applying the AP clustering algorithm.

In short, we propose a novel scheme to analyze resting state fMRI by constructing the global functional connection network via ASR and identifying sub-networks via AP. It is worthwhile to highlight the following features of the proposed scheme: (a) Compared with the conventional bivariate analysis, ASR is a multivariate method which relates one single node with all the other nodes. As a result, it simultaneously considers the influence of all nodes in constructing a global connection network represented by the association matrix. The adaptivity of ASR provided by the trace LASSO regularizer makes ASR a suitable approach for dealing with highly correlated and sparse fMRI data. (b) It uses AP to group the obtained global network into several non-overlapping sub-networks, identifying the connectivity patterns for fMRI data. It obviates the need of setting a threshold on the association matrix.

The rest of this paper is organized as follows. In Section 2, we present the approach to constructing the association matrix via ASR and identifying sub-networks via AP, followed by the experimental setting and description. In Section 3, the experimental results are reported. For testing the proposed scheme, both simulated and real fMRI data are used in the experiment. The Pearson correlation and the ASR technique are compared on both the levels of constructing the association matrix and identifying sub-networks based on AP. Besides, the reliability of ASR and the Pearson correlation is investigated. Then, we discuss the experiment, including limitations and potential usage of the proposed scheme in Section 4. Finally, Section 5 concludes the paper.

## 2. Materials and methods

### 2.1. Notations

Matrices and vectors are represented by upper-case and lower-case letters, respectively. For a vector **v**∈ℝ^*d*^, *Diag*(**v**)∈ℝ^*d*×*d*^ is a diagonal matrix with **v** as its diagonal elements. For a matrix **M**∈ℝ^*d*×*n*^, ||**M**||_*_ denotes the trace norm that sums up the singular values of **M**, and ||**M**||_op_ denotes the operator norm that is the maximum singular value of **M**.

### 2.2. Adaptive sparse representation

Generally, a sparse representation problem is to represent a *d*-dimensional sample **y** using all samples in a dictionary **X**∈ℝ^*d*×*n*^ with an *n*-dimensional sparse solution ***w***. For data with noise, given a tolerance ε > 0, the problem can be formulated as (Wright et al., 2009)

(1) $$\min\|w{\|}_{0},\;\;s.t.\;\;\|\text{y}-\text{X}w{\|}_{2}\leq \varepsilon .$$

However, such ℓ_0_-norm minimization problem is NP-hard (Amaldi and Kann, 1998). In practice, it could be relaxed by replacing the ℓ_0_-norm with the ℓ_1_-norm, given by

(2) $$\min\|w{\|}_{1},\;\;s.t.\;\;\|\text{y}-\text{X}w{\|}_{2}\leq \varepsilon .$$

However, the ℓ_1_-norm suffers from instability when dealing with highly correlated data, since it is prone to randomly choose one sample from all the correlated ones Grave et al. (2011). This suggests that such ℓ_1_-norm-based sparse representation is not very suitable for fMRI data which are often highly correlated between spatially neighboring brain regions. In contrast to the ℓ_1_-norm that pursues parsimonious representation, the ℓ_2_-norm, on the other hand, uses all the samples for the linear representation, which leads to blindness to the exact correlation structure. It is desired to automatically model the correlation structure. In other words, it is beneficial to combine the advantage of the ℓ_1_-norm in variable selection and the advantage of the ℓ_2_-norm in stable behavior for correlated variables. The trace LASSO is therefore developed, which is defined as

(3) $$\|\text{XDiag}(w){\|}_{*}.$$

It has been proved that the ℓ_1_-norm and the ℓ_2_-norm are two extreme cases of the trace LASSO in the sense (Grave et al., 2011)

(4) $$\|w{\|}_{2}\leq \|\text{XDiag}(w){\|}_{*}\leq \|w{\|}_{1},$$

where each column of **X** is normalized to unit norm. Specifically, when column samples in **X** are identical (i.e., the extreme case of highly correlated data), the trace LASSO becomes the ℓ_2_-norm, while when samples in **X** are orthogonal (i.e., the extreme case of uncorrelated data), the trace LASSO turns out to be the ℓ_1_-norm. Trace LASSO brings both the sparsity of the ℓ_1_-norm and the grouping effect of the ℓ_2_-norm.

By using the trace LASSO as regularizer in the linear representation, the ASR is formulated as

(5) $$\underset{w}{\min}\|\text{XDiag}(w){\|}_{*},\;\;s.t.\;\;\|\text{y}-\text{X}w{\|}_{2}\leq \varepsilon .$$

The trace LASSO adaptively mediates between the ℓ_1_-norm and the ℓ_2_-norm. It behaves like the ℓ_1_-norm for almost uncorrelated variables and like the ℓ_2_-norm for strongly correlated variables. The optimization (Equation 5) can be converted into

(6) $$\underset{w}{\min}\frac{1}{2}\|\text{y}-\text{X}w{\|}_{2}^{2}+\lambda \|\text{XDiag}(w){\|}_{*},$$

where λ > 0 is a regularization parameter. For the choice of λ, the initial value giving the upper bound of λ can be obtained according to the formula given by Grave et al. (2011), i.e.,

(7) $$\lambda =\|\text{X}{\|}_{\text{op}}\|{\text{X}}^{\text{T}}\text{y}{\|}_{\infty }.$$

When λ achieves this upper bound, the most sparse solution 0 will be obtained. As λ decreases, the solution will become less sparse. Thus, to search for an appropriate λ, we could start from this upper bound and then decrease its values gradually. That is, the solution becomes denser gradually from the trivial zero solution until reaching optimality (Grave et al., 2011). This optimization problem (Equation 6) can be solved by Alternating Direction Method (ADM), where a globally optimal solution is achieved, as used by Lu et al. (2013) in studying subspace segmentation.

### 2.3. Association matrix construction with ASR

Let X = [x_1_, …, x_*n*_] ∈ ℝ^*d*×*n*^ be the fMRI data matrix, where *n* denotes the number of nodes and *d* the number of time points. Suppose that **x**_*i*_ has been normalized. For the vector **x**_*i*_, its corresponding dictionary for sparse representation consists of all the nodes except for itself, i.e., X_*i*_ = [x_1_, …, x_*i*−1_, x_*i*+1_, …, x_*n*_] ∈ ℝ^*d*×(*n*−1)^. Then the calculation of the association of **x**_*i*_ with all other nodes by ASR boils down to

(8) $$\underset{w_{i}}{\min}\frac{1}{2}\|{\text{x}}_{i}-{\text{X}}_{i}w_{i}{\|}_{2}^{2}+\lambda \|{\text{X}}_{i}\text{Diag}(w_{i}){\|}_{*},$$

where $w_{i}\in {\mathbb{R}}^{n-1}$ is a coding coefficient vector corresponding to **x**_*i*_. We pad ***w***_*i*_ with a zero in the *i*th position, denoted by ${\tilde{w}}_{i}\in {\mathbb{R}}^{n}$, which means the association between **x**_*i*_ and itself. The *j*th element of ${\tilde{w}}_{i}$ represents the association between **x**_*i*_ and **x**_*j*_. Stacking all the coefficient vectors ${\tilde{w}}_{i}$ results in the coefficient matrix $\tilde{\text{W}}=[{\tilde{w}}_{1},\ldots ,{\tilde{w}}_{n}]\in {\mathbb{R}}^{n\times n}$.

We usually prefer a symmetry and non-negative association matrix **A** for fMRI functional connectivity. For such purpose, we could replace $\tilde{\text{W}}$ with $\text{A}=(|\tilde{\text{W}}|+|\tilde{\text{W}}{|}^{\text{T}})/2$. Each element *a*_*ij*_ in **A** represents the connection strength between node *i* and node *j*, and all diagonal elements *a*_*ii*_ = 0.

**Figure 1:**
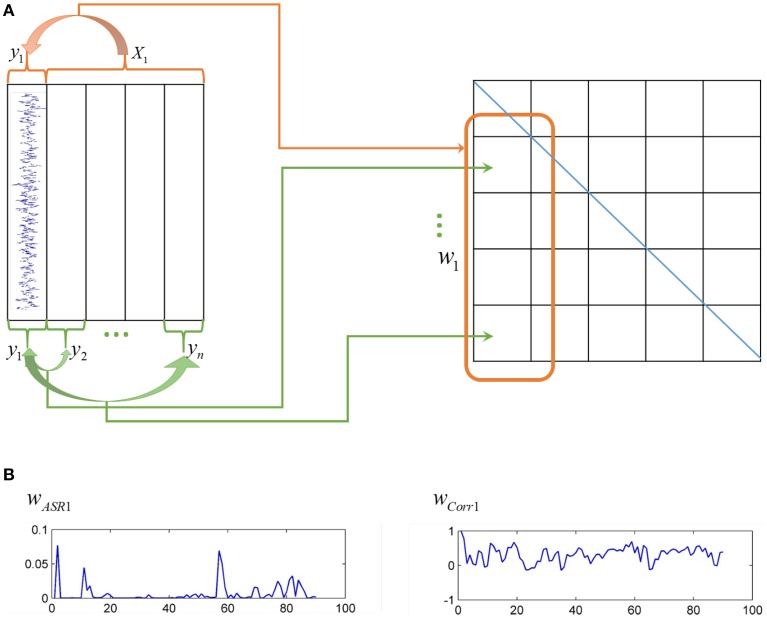
**Different ways of ASR and the Pearson correlation to compute association matrices**. **(A)** On the left panel, each column represents time series of each node, while on the right panel, each grid represents an element of an association matrix. The Pearson correlation computes the association between pairwise nodes as shown by the green arrows, while ASR considers the association between one node and all other nodes simultaneously as shown by the red arrow. **(B)** The left panel is an illustration of one typical column of the association matrix (i.e., the association of one node with all other nodes) derived by ASR, while the right panel derived by the Pearson correlation.

Actually, correlation-based methods and sparse representation-based methods are two distinct ways to construct association matrices of fMRI data, as shown in Figure 1. The Pearson correlation calculates the pairwise association between nodes without considering other nodes' influence. The correlation coefficients are taken as connection strengths. By contrast, sparse representation-based methods obtain the association between one node and all other nodes simultaneously. The sparse coefficients obtained represent connection strengths.

### 2.4. Clustering analysis with AP

After computing the association matrix, the AP clustering algorithm is then employed to identify connectivity patterns by grouping all nodes into distinct sub-networks. The recently developed AP clustering algorithm has attractive advantages over many classical clustering methods (Frey and Dueck, 2007). For example, it does not require prespecifying the number of clusters and initializing clustering centers, and the input to AP could be a general non-metric similarities. Moreover, AP could be simply implemented. In fact, it includes all data points as possible exemplars and controls the number of clusters by adjusting the value of preference for each data point. The input of AP is a similarity matrix with preference values as its diagonal elements, which is usually measured by the Euclidean distance or the Pearson correlation in brain data mapping. In our proposed scheme, the association matrix computed by ASR or the Pearson correlation is taken as the similarity matrix, and are input into the AP algorithm directly. Here, for each individual computation, a common preference value is assigned to all nodes, which means all data points are equally treated as exemplars without using any prior knowledge.

### 2.5. fMRI data sets

To test our proposed scheme, we perform experiments on both simulated and real resting state fMRI data sets. The simulated data sets, generously provided by Smith et al. (2011), are made available from http://www.fmrib.ox.ac.uk/analysis/netsim/correction.html, where the data set Sim4 is used in our experiment. This data set contains simulated resting state fMRI data of 50 subjects, each with 50 nodes and 200 time points (TR is set as 3 s). The underlying network structure consists of 10 linked clusters with each cluster being a five-node ring, as described in Smith et al. (2011). These data are generated by using dynamic causal modeling (DCM; Friston et al., 2003), and noises are added on both neural and mean signal levels. More detailed information about the simulated data sets can be found in Smith et al. (2011).

The real resting state fMRI data set for experiment is from the Neuroimaging Informatics Tools and Resources Clearinghouse (NITRC) 1000 functional connectomes project (Biswal et al., 2010). A subset of 20 subjects from the data set Beijing_Zang containing 198 subjects are downloaded from the 1000 Functional Connectomes Project online database. Detailed information about this data set can be found in http://fcon_1000.projects.nitrc.org.

Besides, another public real resting state fMRI data set provided by (Mao et al., 2015) is used to investigate the test-retest reliability of connectivity metrics. Ten subjects out of 21 healthy adults from the data set are used, each containing two sessions. Details of this data set are available at http://datadryad.org/resource/doi:10.5061/dryad.4kb75.

### 2.6. Data preprocessing

The real fMRI data are preprocessed by using the Statistical Parametric Mapping package (SPM8) with the Data Processing Assistant for Resting-State fMRI (DPARSF; Yan and Zang, 2010) toolbox implemented in MATLAB R2011b. The first 10 volumes of each subject are discarded. The preprocessing steps for the remaining 215 volumes include the following items: (a) slice timing, (b) realignment, (c) regressing out of the six motion parameters, whole brain, cerebrospinal fluid and white matter signals, (d) spatial normalization to MNI space by DARTEL procedure and resampling them to the voxel size of 3 × 3 × 3 mm, and (e) spatial smoothing with a 4 mm full width half maximum (FWHM) Gaussian kernel and filtering using a bandpass filter (0.01–0.1 Hz). The data set for testing reliability is preprocessed in the same way, except that the step of spatial normalization to MNI space is carried out by using EPI templates due to the absence of T1 images. For both data sets, no subject is excluded under the criteria that head motion is less than 2 mm of translation or 2° of rotation in any direction. Then for each session of each subject, time series of 90 ROIs are extracted by using the AAL (Tzourio-Mazoyer et al., 2002) template, resulting in a data matrix of 215 (225 for the reliability data set) time points by 90 brain ROIs. Here, with the aim to ensure comparability, the AAL template is used to define nodes, as adopted in most studies (Liu et al., 2008; Ferrarini et al., 2009; He et al., 2009; Braun et al., 2012; Ryali et al., 2012).

### 2.7. Data analysis

**Figure 2:**
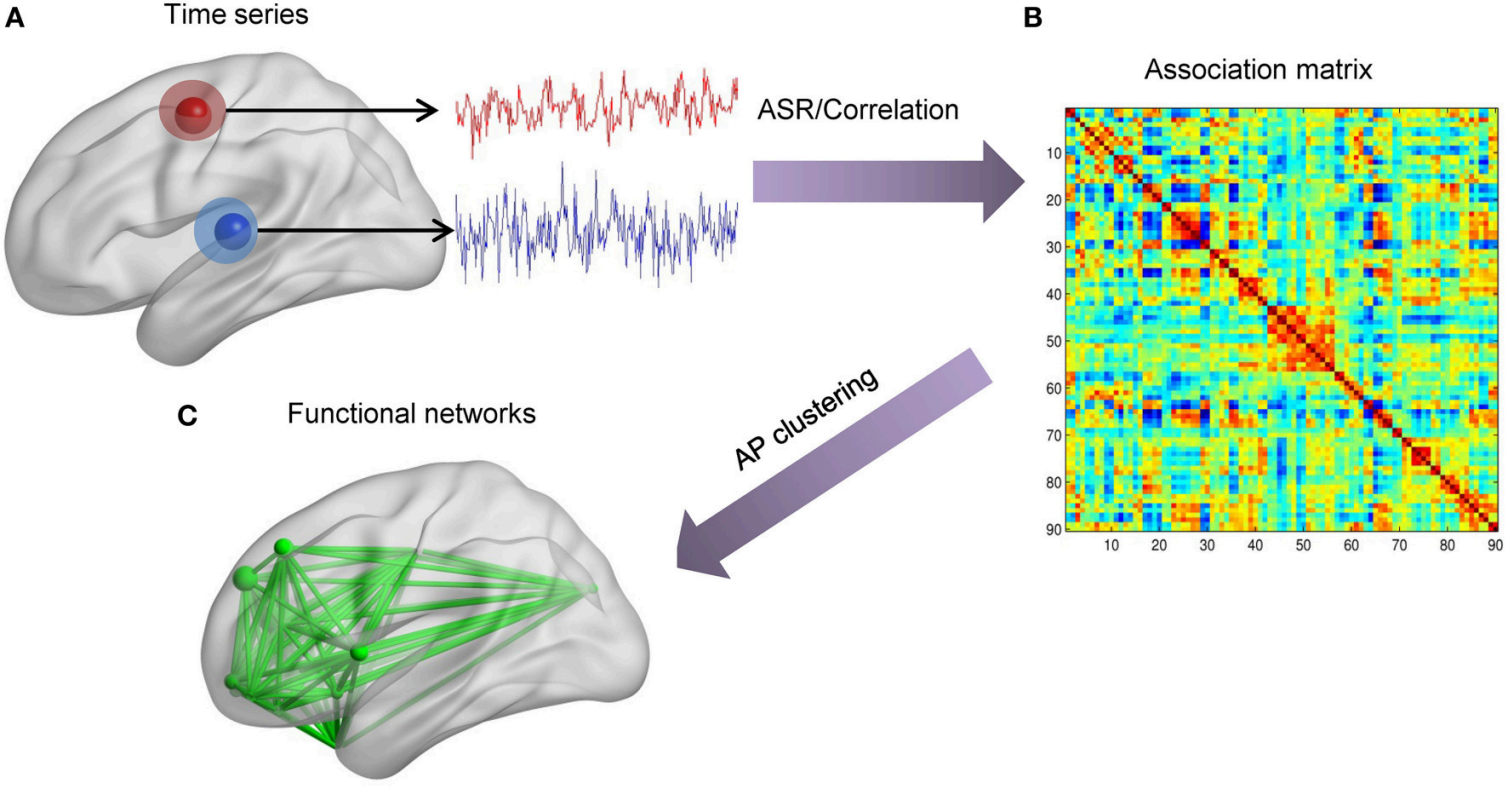
**The scheme of identifying brain functional networks**. **(A)** Extract time series of each node defined by the AAL template. **(B)** Use ASR or the Pearson correlation to compute the association matrix. **(C)** Identify sub-networks by using the AP clustering algorithm based on the association matrix.

For simulated and the first real fMRI data sets, the scheme of data analysis mainly contains two parts, as shown in Figure 2. Firstly, we extract time series of each node. Then, for each individual, the association matrix is computed via the ASR or the Pearson correlation based on the normalized fMRI time series. Consequently, for each individual, we obtain ASR- and correlation-driven global networks. Secondly, the AP clustering algorithm is used to group these global networks into smaller distinct sub-networks, thus identifying connectivity patterns for each individual. The initial value of λ is calculated according to Equation (7), and a wide range of λ values are tested for the both data sets. Specifically, the optimal λ is selected for each subject based on their performance evaluation metrics as well as convergence criteria. The initial values of λ for all samples are around 1. So, λ is initially set to vary from 1 to 10^−4^ with the step of the logarithm values being −1. We then refine the search in a narrowed range from 0.3 to 0.1 with a step size of −0.01. As will be seen, most of the optimal λ values are around 0.2. Likewise, for the real fMRI data set, the initial values of lambda are around 5. So, it is initially set to vary from 5 to 5 × 10^−4^ with the step of the logarithm values being −1, and then is refined to the range from 1 to 0.1 with a step size of −0.1. Based on this search, the final value of λ is set as 0.5 for all subjects.

On the data set for testing reliability, both ASR and the Pearson correlation are used to obtain association matrices, based on which a reliability measure is computed. For a comprehensive investigation of the test-retest reliability of ASR, six ASR-driven association matrices for each session of each subject are estimated with λ being 1, 0.5, 0.1, 0.01, 0.001, and 0.0001 where the initial value of λ is set around 5.

### 2.8. Evaluation metrics

An advantage of the experiment on the simulated data set is that we could compare the experimental results with the ground truth (known beforehand) of both the global connection matrix and the network structure after clustering. Because the ground truth of the global connection matrix given in Smith et al. (2011) is directed while the association matrix obtained in our experiment by either the ASR or the Pearson correlation does not contain any direction information, a sensitivity measure is used to evaluate the ability of different approaches to separating true positive (TP) connections from false positive (FP) connections. The sensitivity measure is defined as

(9) $$sen=\frac{\#\{\text{TP}>95th\%(\text{FP})\}}{\#\{\text{TC}\}},$$

which calculates the proportion of the number of the TP connection strengths that are larger than the 95th percentile of the FP connection strengths. Here, #{TC} denotes the number of all true connections as in the ground truth. That is, #{TC} = #{TP} + #{FN}, where FN denotes false negative connections. Equation (9) is evaluated on the simulated data set, where the estimated global functional connections are reflected in the association matrix. The non-zero values of the association matrix indicate connections between corresponding nodes. Then, the discovered connections are compared with the ground of truth. The TP mean that the discovered connections are truly existed while the FP connections are in fact not existed according to the ground truth. Note that Equation (9) is the same as the measure “c-sensitivity” in Smith et al. (2011), and we use the same approach to measure sensitivity, TP and FP as in Smith et al. (2011). For evaluating the clustering performance, after clustering all ASR and the Pearson correlation matrices into around 10 clusters by AP, the Hungarian algorithm (Lovász and Plummer, 1986) is then used to match the clustering labels with the ground truth labels. Then the clustering accuracy is computed as (Zheng et al., 2004)

(10) $$acc=\frac{\sum_{i=1}^{n}\delta (g_{i},c_{i})}{n},$$

where *g*_*i*_ and *c*_*i*_ are the labels of the *i*th node of the ground truth and the clustering result after matched by the Hungarian algorithm, respectively. Note that δ(*g*_*i*_, *c*_*i*_) equals to one if and only if *g*_*i*_ = *c*_*i*_, and zero elsewhere. Simply put, it counts the number of nodes that have the same labels with the ground truth labels.

For the first real fMRI data set, two quantifiable indexes are used as evaluation criteria without knowing the ground truth. By using the Brain Connectivity Toolbox (BCT; Rubinov and Sporns, 2010), a network measure, modularity, is computed to investigate the community structure of the global functional connection network for each association matrix. The modularity index measures the quality of the division of nodes. It favors the division that has highly connected nodes within sub-groups but sparsely connected nodes between sub-group networks (Newman, 2006). The number of sub-groups (i.e., communities) obtained by such division is also recorded for each association matrix. Another index, Silhouette, is used to measure the quality of clustering (Zhang et al., 2011), where different levels of clustering (i.e., different numbers of clusters) are tested. The Silhouette value of a node is computed by using the following formula (Rousseeuw, 1987)

(11) $$s(i)=\frac{a(i)-b(i)}{\max\{a(i),b(i)\}},$$

where *a*(*i*) denotes the average similarity between node *i* and the other nodes that are in the same cluster with node *i*, and *b*(*i*) represents the biggest one of all the average similarities between node *i* and the nodes of another clusters. In this scenario, similarities are defined as the association between nodes computed by either ASR or the Pearson correlation. The average Silhouette value *s* over all nodes can be used as an index measuring the quality of a clustering result. The higher the value *s*, the better the clustering quality. Since Equation (11) is variant with translation, for fair comparison, we perform a preprocessing of the association matrices before applying Equation (11). Specifically, we require the association matrices produced by ASR and the Pearson correlation to have the same level of magnitude. For this purpose, we translate the entries of the association matrices such that they have the same value of the global mean (i.e., the average of all the entries of each association matrix). Note that Equation (11) is invariant with rescaling.

On the data set for evaluating the test-retest reliability, a measure for comparing ASR and the Pearson correlation is quantified by the intra-class correlation coefficient (ICC; Shrout and Fleiss, 1979), as used in many researches (Zuo et al., 2010; Braun et al., 2012; Cao et al., 2014; Zuo and Xing, 2014). The ICC index used in this paper adopts the two-way mixed model for single consistency, given by

(12) $$\text{ICC}(C,1)=\frac{MS_{B}-MS_{E}}{MS_{B}+(k-1)\;*\;MS_{E}},$$

where *MS*_*B*_, *MS*_*E*_, and *k* denote the between-subjects mean square, the error mean square, and the number of repeated sessions, respectively.

## 3. Results

### 3.1. Results on simulated data set

**Figure 3:**
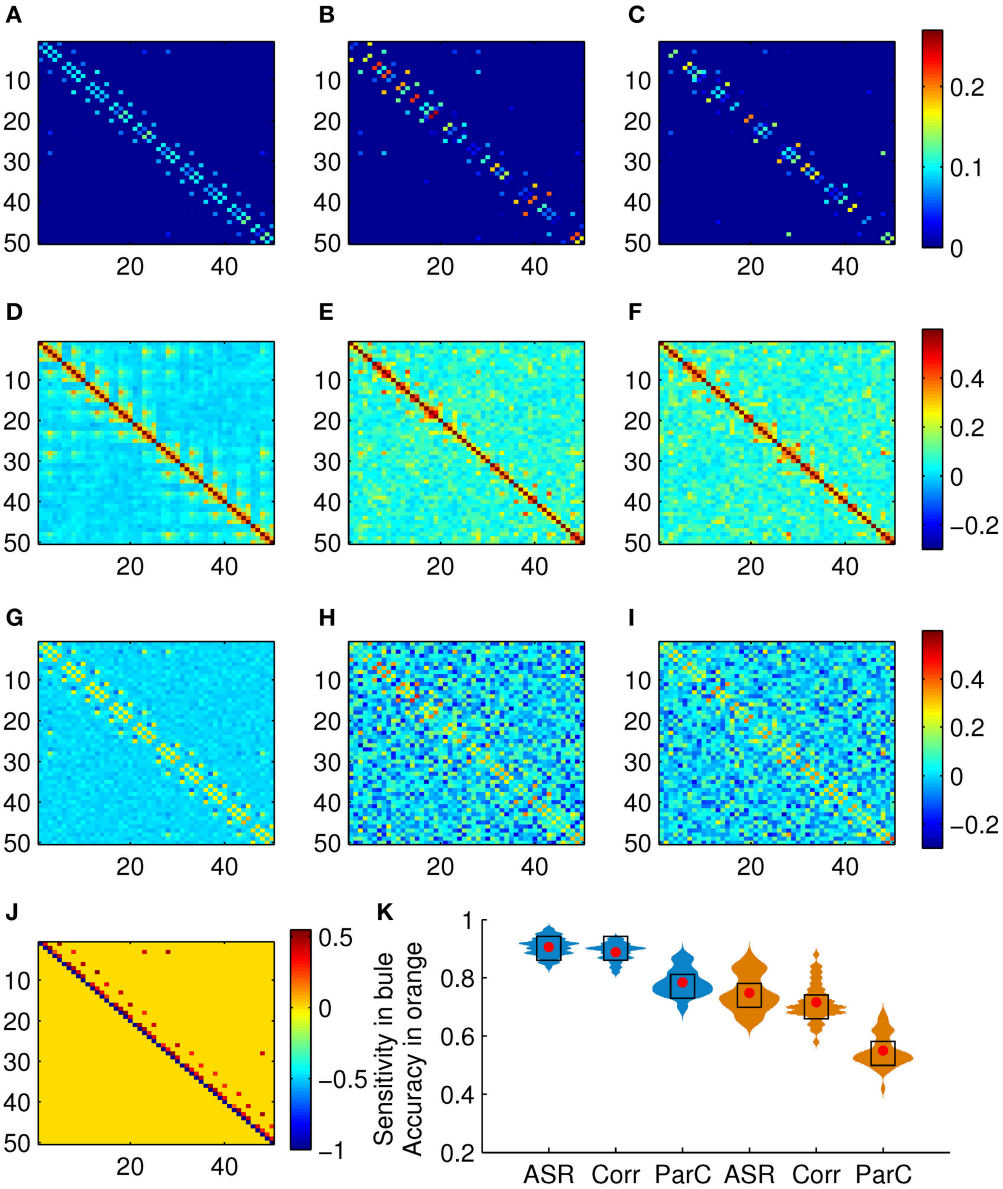
**Results on the simulated data set**. **(A)** Mean association matrix averaged over 50 subjects by using ASR. Panels **(B,C)** are association matrices derived from ASR on two randomly selected subjects. **(D)** Mean association matrix averaged over 50 subjects by using the Pearson correlation. Panels **(E,F)** are the corresponding association matrices derived from the Pearson correlation on the two randomly selected subjects. **(G)** Mean association matrix averaged over 50 subjects by using the partial correlation. Panels **(H,I)** are the corresponding association matrices derived from the partial correlation on the two randomly selected subjects. **(J)** Ground truth of connection matrix. **(K)** Sensitivity (drawn in blue) and clustering accuracy (drawn in orange) distributions (with width denoting frequency) of ASR, the Pearson correlation, and the partial correlation over 50 subjects, where red dots and black blocks represent the mean and the median respectively.

We test the proposed scheme on the simulated data set of 50 subjects. The performance is evaluated in terms of sensitivity and clustering accuracy. Figure 3 shows the association matrices produced by ASR, the Pearson correlation, and the partial correction, as well as the ground truth matrix, and the distributions of the sensitivity and clustering accuracy. The clustering results delineated in Figure 3H are obtained on this simulated data with the known ground truth of 10 clusters. To compare the clustering accuracies of the three methods, in this experiment, we adjust the preference value in the AP algorithm and expect to obtain 10 clusters (for only a few subjects, the AP algorithm may not converge to 10 clusters, but it will result in a very close number around 10, say 9 or 11). We point it out that, given the initial parameters of the AP algorithm, the cluster membership is determined automatically and can not be manipulated subjectively. Then the Hungarian algorithm is used to match the clustering labels with the ground truth labels. Finally, the clustering accuracy is calculated by Equation (10). In this sense, the clustering results delineated in Figure 3H are comparable.

It is observed from Figure 3H that the partial correlation yields rather poor performance in terms of the measures of sensitivity and accuracy with the mean reaching 78.43 and 55.00%, respectively. The partial correlation still estimates the dependency between a pair of nodes, even though it removes possible linear influence of other nodes. In the following experiments, we only investigate the performances of ASR and the Pearson correlation. The reasons we choose the Pearson correlation for comparison is that it is one of the most widely used methods of estimating functional connectivity due to its simplicity and efficiency and it could be served as a representative of bivariate methods.

Two-sample *t*-tests show that both the sensitivity and the clustering accuracy of ASR are significantly higher than those of the Pearson correlation (*p* < 0.001). It is seen that both ASR and the Pearson correlation demonstrate good performance in terms of the sensitivity, with the mean sensitivity reaching 90.59 and 88.82%, respectively. However, the ASR approach is significantly more capable of identifying TP connections and separating them from FP ones than the Pearson correlation. The mean clustering accuracy of ASR and the Pearson correlation are 74.84 and 71.56%, respectively. Still, ASR performs significantly better than the Pearson correlation in capturing the underlying network structure delineated by connection strengths.

### 3.2. Results of estimating global functional connection network

**Figure 4:**
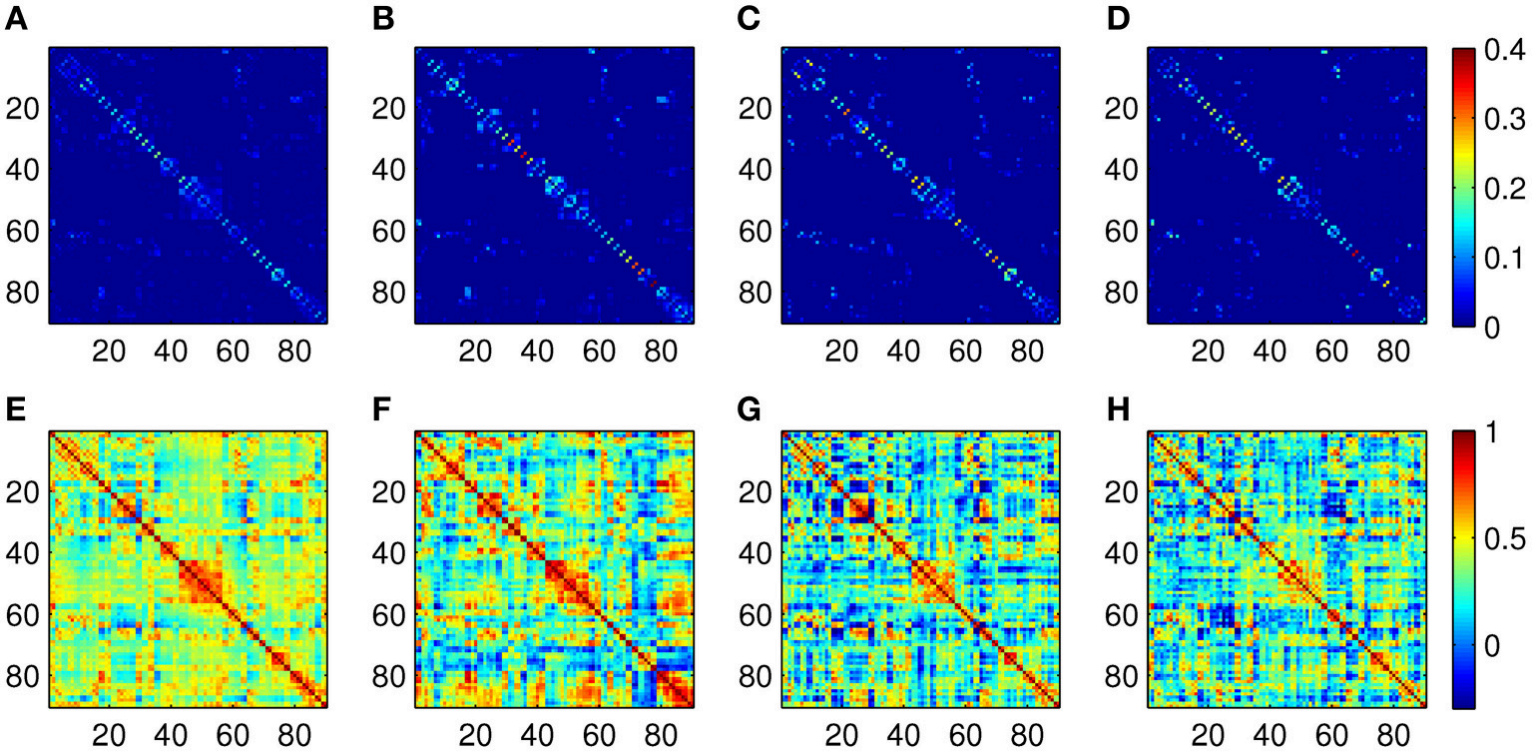
**Examples of association matrices computed by using ASR and the Pearson correlation**. **(A)** Mean matrix averaged over 20 subjects by using ASR. Panels **(B–D)** are association matrices derived from ASR on three randomly selected subjects. **(E)** Mean matrix averaged over 20 subjects by using the Pearson correlation. Panels **(F–H)** are corresponding association matrices derived from the Pearson correlation on the above three subjects respectively.

The Pearson correlation and the proposed ASR schemes are then applied to the real resting state fMRI data set. Some examples of the 90-node association matrices obtained by the two methods are illustrated in Figure 4. It is observed from Figure 4 that ASR achieves better sparsity than the Pearson correlation in both individual matrices and the mean matrix. In other words, ASR leads to a more sparsely connected functional network in contrast to the Pearson correlation.

The quality of community structure, as measured by the modularity index, of these global functional networks are also investigated for each subject. A two-sample *t*-test for the modularity of all the 20 subjects shows that the modularity driven by ASR (mean = 0.50 ± 0.12) is significantly higher than that of the Pearson correlation (mean = 0.13 ± 0.06; *p* < 0.001). In other words, compared with the Pearson correlation, the ASR-driven networks achieve better quality of community structure. The number of communities also yields a big difference between ASR (median = 7) and the Pearson correlation (median = 3).

**Figure 5:**
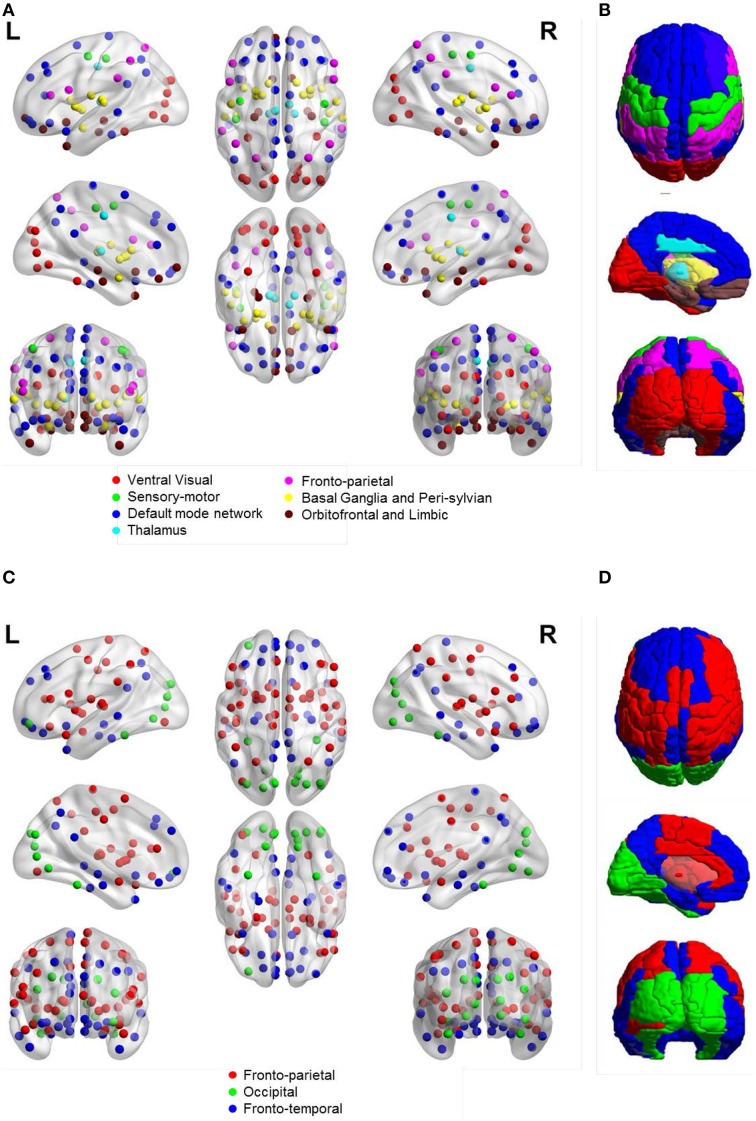
**Community structure of a functional network obtained by ASR and the Pearson correlation on a randomly chosen subject**. **(A)** ASR with nodes shown. **(B)** ASR with ROIs shown. **(C)** Pearson correlation with nodes shown. **(D)** Pearson correlation with ROIs shown. We show the views of axial, coronal and sagittal for left and right hemispheres. Ninety nodes are shown, where each node represents a ROI in AAL template and nodes of the same color form one community. This figure is generated by using BrainNet Viewer (Xia et al., 2013).

Figure 5 shows the community structure of a functional network obtained by ASR and the Pearson correlation on a randomly chosen subject. As shown in Figure 5, seven communities are revealed by ASR, including cortices of ventral visual, sensory-motor, default mode network (DMN), thalamus, fronto-parietal, basal ganglia with peri-sylvian and orbitofrontal with limbic. One notable feature suggested by these findings is that the functional network structure delineated by ASR is tightly connected within a community while sparsely connected between communities. By contrast, the network obtained by the Pearson correlation consists of three large communities consisting of fronto-parietal, occipital and fronto-temporal networks, all of which involve many brain areas with different functions. Thus, the community structure is difficult to be interpreted.

### 3.3. Results of AP clustering analysis

**Figure 6:**
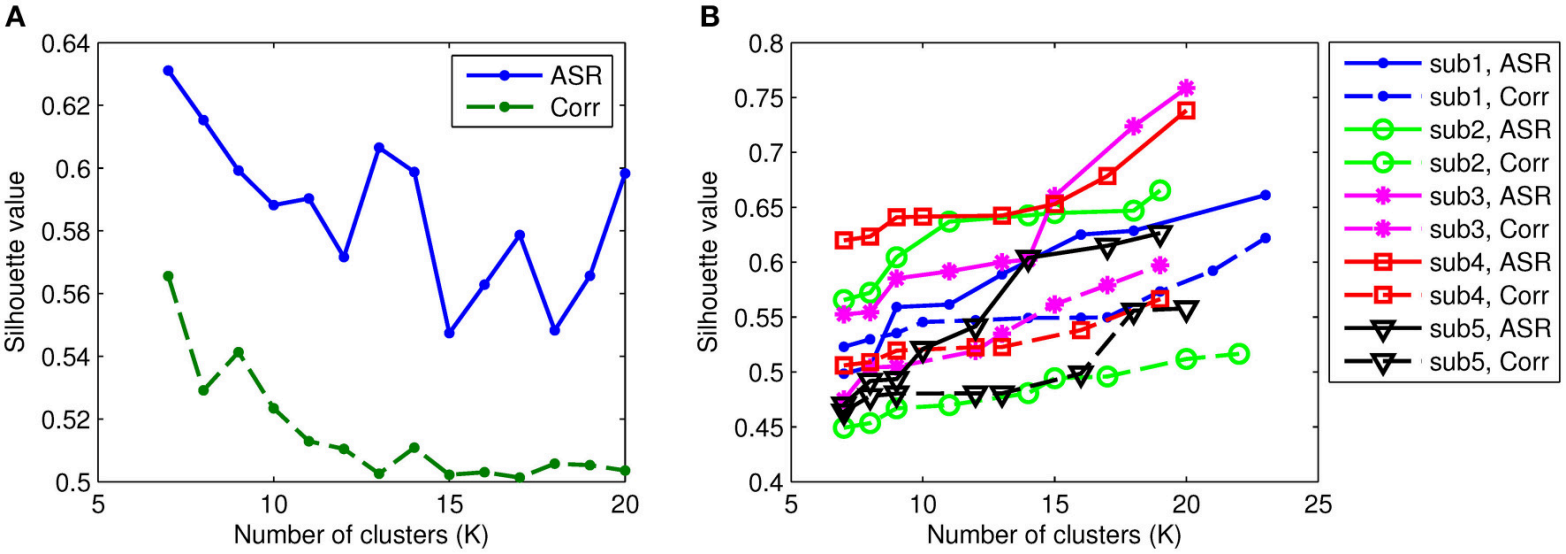
**Silhouette values of ASR and the Pearson correlation with varying numbers of clusters**. Each colored line represents the results of one subject. The solid lines illustrate the Silhouette performance of the AP clustering results based on ASR while the dashed lines based on the Pearson correlation. **(A)** Mean Silhouette values. **(B)** Examples of Silhouette values on five randomly selected subjects.

To identify functional connectivity patterns, the AP clustering algorithm is then applied to the obtained association matrices for each individual. The AP algorithm does not prespecify the number of clusters explicitly. Rather, the number of identified clusters is controlled by the input values of preferences for the data points and the iterative procedure of message-exchanging. In our experiments, we adjust the preference values for both ASR and Pearson's correlations such that they obtain comparable numbers of clusters. For example, we group ASR and correlation matrices for all subjects into 10 clusters by assigning different preference values with the AP algorithm. So, the parameters used for ASR and Pearson's correlations can be different. We investigate the number of clusters, denoted by *K*, through 7 to 20 (when *K* reaches above 20, the AP algorithm does not converge or groups one single node into a cluster). This is worked as setting the preference value. In the following, for simplicity, we use ASR to represent ASR plus AP procedures, and so is the Pearson correlation. The quantifiable index, Silhouette value, is computed to assess the resulting clustering quality for each subject. Figure 6 compares the Silhouette values of both ASR and the Pearson correlation with different clustering levels. As can be seen, the mean Silhouette value of ASR is always much higher than that of the Pearson correlation for any clustering number. Note that with the AP clustering algorithm the resulting number of clusters on a specific subject may differ from the initially set number. The mean Silhouette value is calculated over the clustering results with the same number of clusters. We also in Figure 6 demonstrate the Silhouette values on five randomly selected subjects. It clearly shows that the Silhouette values of ASR are much higher than that of the Pearson correlation for all the selected subjects. Two-sample *t*-tests reveal that the Silhouette values of ASR are significantly higher than that of the Pearson correlation (*p* < 0.001) regardless of the number of clusters. It indicates that on the same level of clustering (i.e., the same clustering numbers), the clustering quality of ASR is substantially and stably better than that of the Pearson correlation.

**Figure 7:**
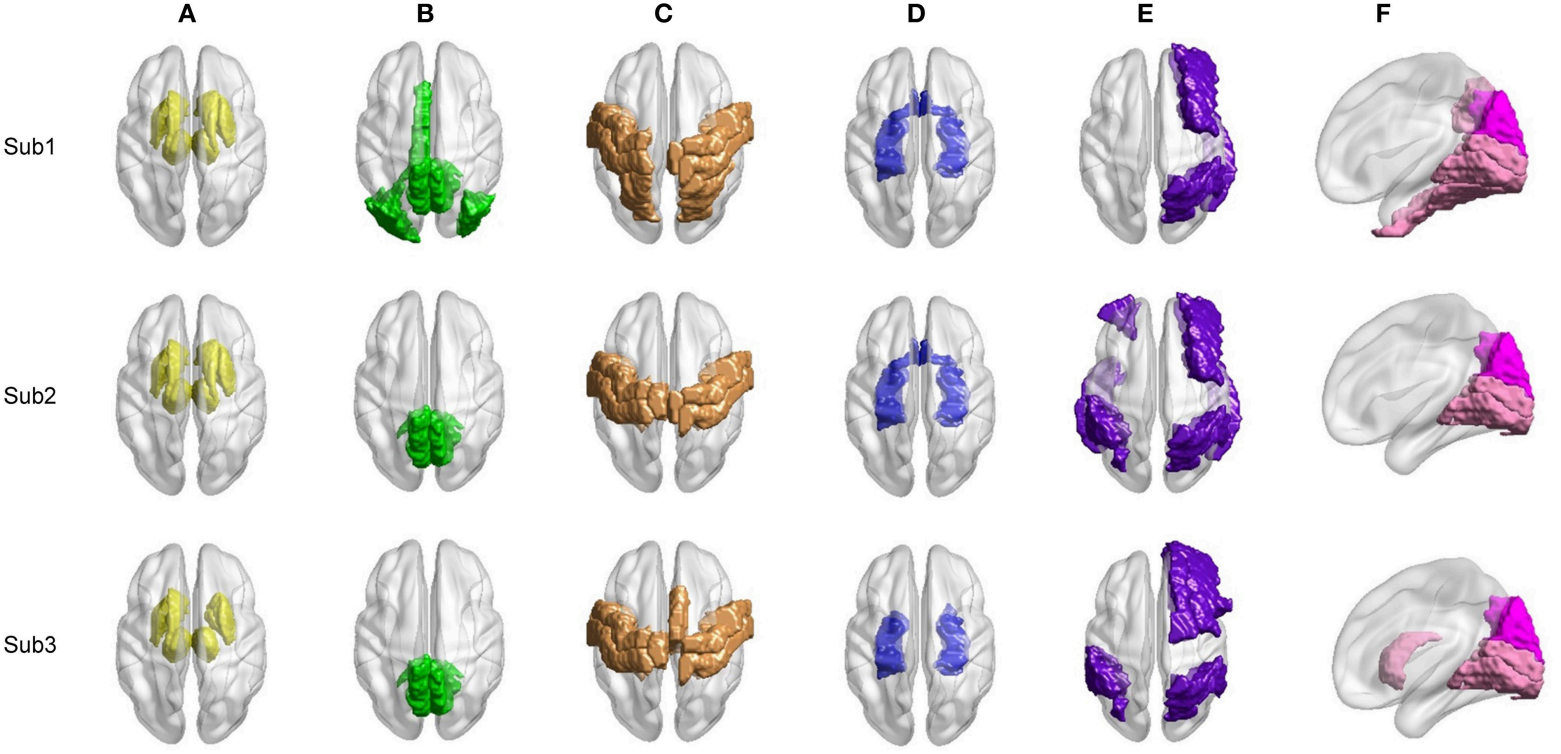
**Examples of six RSNs on three randomly selected subjects**. **(A)** Basal ganglia. **(B)** Main parts of DMN including precuneus and PCC. **(C)** Sensorimotor cortex. **(D)** Limbic. **(E)** Frontal-parietal network. **(F)** Visual cortex including ventral visual and dorsal visual sub-networks. Regions marked in the same color belong to the same network.

**Figure 8:**
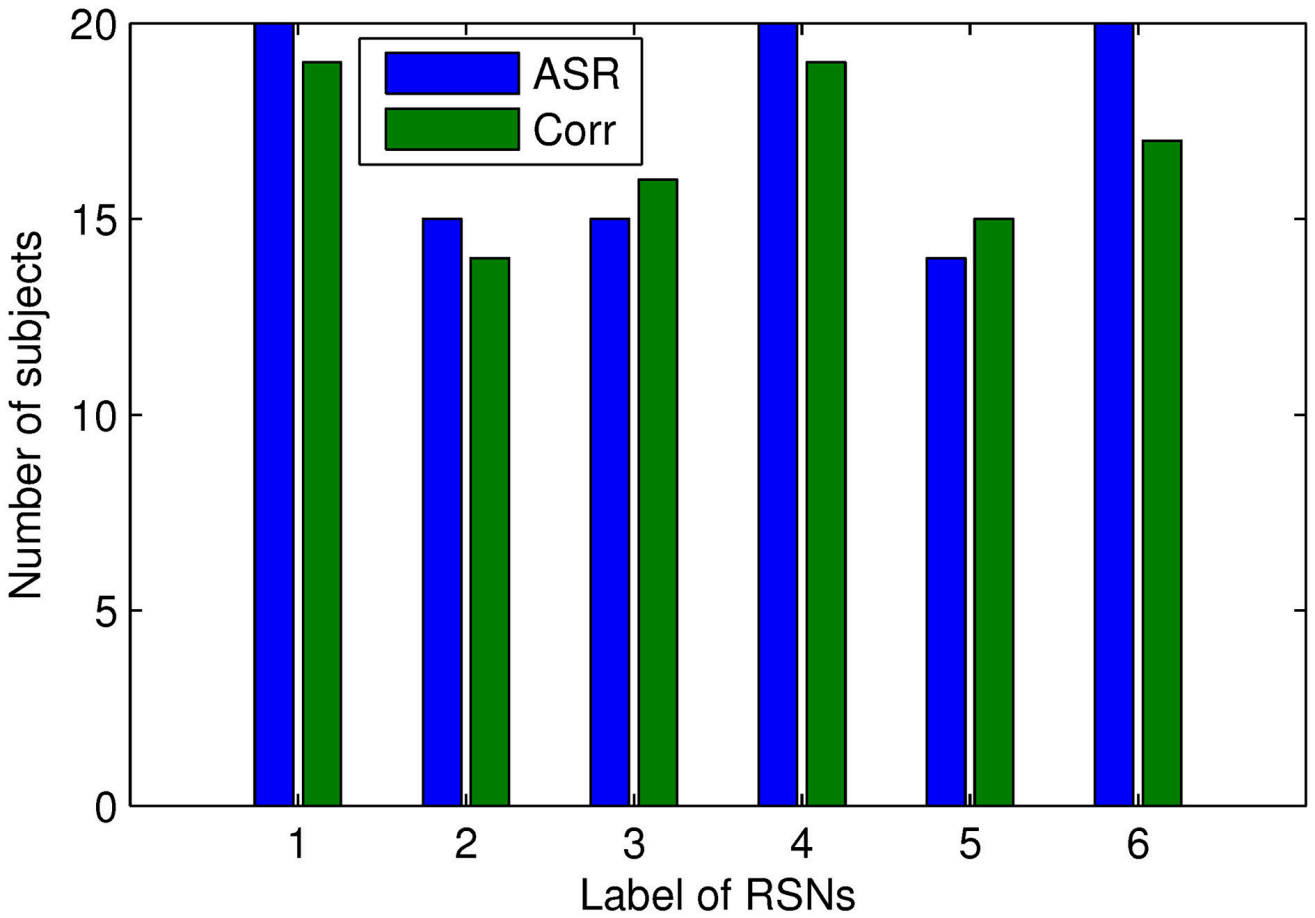
**Number of subjects reporting the presence of the above 6 RSNs**. The digits “1” through “6” denote Basal ganglia, DMN, sensorimotor cortex, limbic, frontal-parietal network, and visual cortex respectively. Results of ASR and the Pearson correlation are marked in blue and green respectively.

We proceed to analyze the connectivity patterns identified by AP from the viewpoint of neurophysiology. We first compare the connectivity patterns driven by ASR (followed by AP) and resting state networks (RSNs) commonly reported in previous studies (van den Heuvel and Pol, 2010, and references therein). Figure 7 shows some examples of clustering results on three randomly chosen subjects, where six main networks out of fifteen are drawn. As shown in Figure 7, although regions within each sub-network are not exactly matched between different subjects, key regions for a specific function are grouped into a same sub-network. For example, the DMN mainly includes precuneus and posterior cingulate cortex (PCC), which are two main parts of DMN (Fransson and Marrelec, 2008). The frontal-parietal network mainly includes superior frontal regions and superior parietal regions (Mantini et al., 2007). Figure 8 displays the frequency of each sub-network reported in the results of all the 20 subjects for both ASR and the Pearson correlation. As shown in Figure 8, most subjects report the six networks mentioned above, and ASR yields slightly more networks than the Pearson correlation in general. The RSNs extracted by ASR are more consistent with RSNs reported in previous studies, implying that ASR is a reasonable method for analyzing resting state fMRI data.

**Figure 9:**
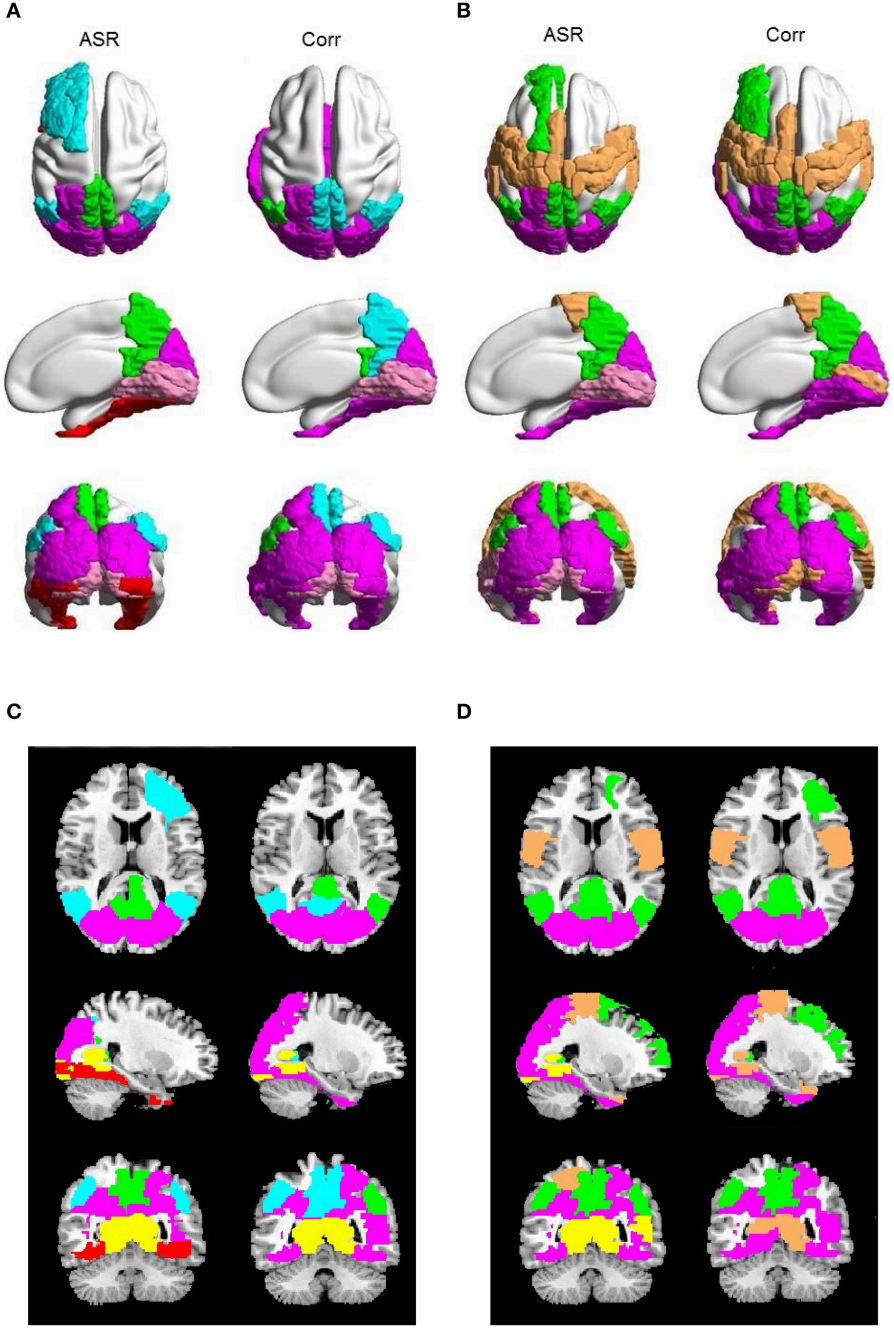
**Examples of saliently different sub-networks between ASR and the Pearson correlation on one randomly selected subject**. In each panel, the results of ASR are drawn on the left side and the Pearson correlation the right side. **(A)** The clustering number *K* is 15. **(B)** The clustering number *K* is 10. Each color represents a sub-network. **(C,D)** are the corresponding 2D rendering of **(A,B)**, respectively.

Then for each subject, we compare ASR and the Pearson correlation from the perspective of the details of sub-networks. The most significant difference is that, for each subject at each clustering level, ASR often tends to achieve a better parcellation which is easier to be interpreted. Figure 9 shows examples of some sub-networks that embody salient difference between ASR and the Pearson correlation on one randomly selected subject. Specifically, Figure 9A mainly shows two sub-networks including DMN and the visual network, where the parcellation is relatively fine-grained. Compared with the Pearson correlation, ASR divides larger networks into smaller and meaningful ones in which regions are functionally tightly correlated. For example, DMN in ASR is divided into two parts: one part is the core regions of DMN including precuneus and PCC and the other part includes angular. While in the Pearson correlation, precuneus and PCC are scattered into two different sub-networks. Furthermore, ASR divides the visual network into three parts including the primary visual cortex, the dorsal pathway and the ventral pathway while the Pearson correlation fails to capture this feature. Figure 9B mainly shows three sub-networks including DMN, the visual network and the sensorimotor network, where the parcellation is relatively coarse. In this situation, ASR still performs better than the Pearson correlation. DMN in ASR includes precuneus, PCC, angular and part of medial prefrontal cortex, while in the Pearson correlation it only includes precuneus, PCC and the right angular. Besides, ASR divides the visual network into the primary visual cortex and the extra striate visual cortex, while in the Pearson correlation the primary visual cortex is grouped into the sensorimotor network. Although the obtained sub-networks vary from subject to subject, they all suggest that ASR achieves a better clustering quality than the Pearson correlation, which is also consistent with the results of the Silhouette values.

### 3.4. Results of reliability analysis

**Figure 10:**
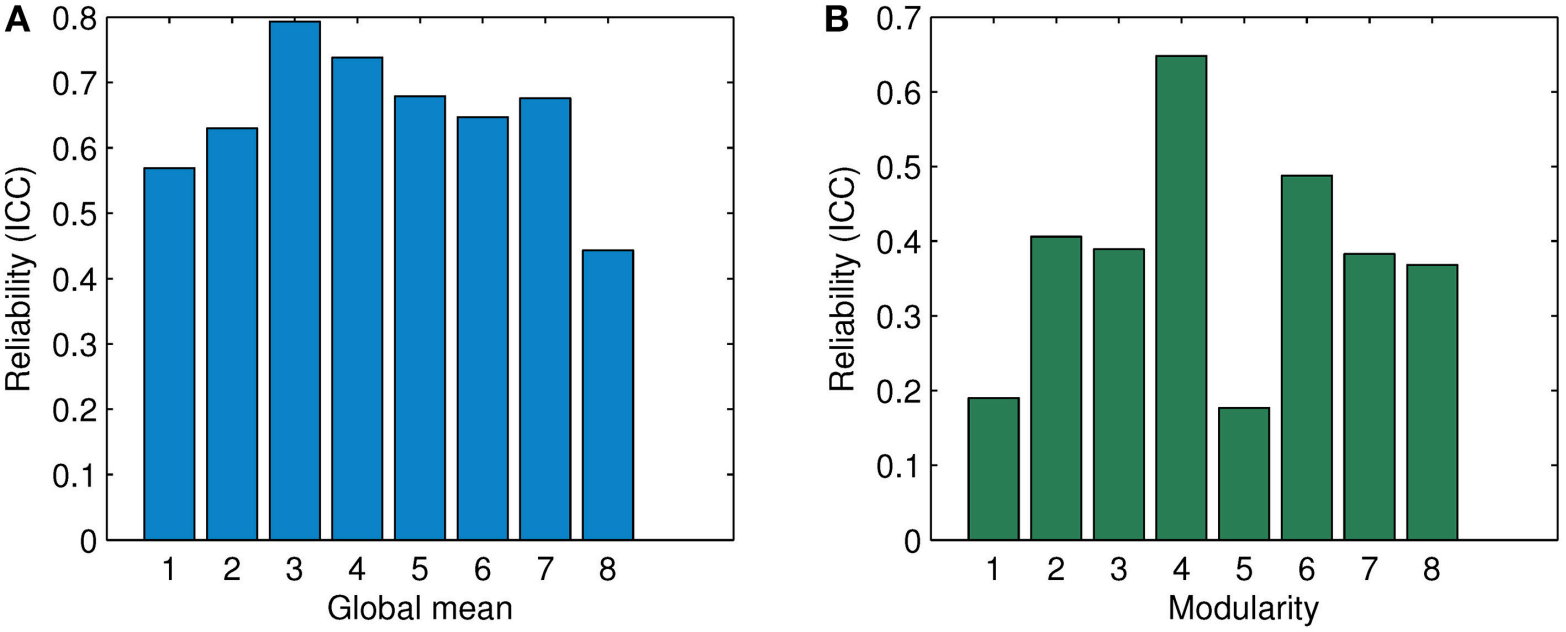
**Comparison of ICC in terms of the global mean and the modularity for association matrices obtained by ASR and the Pearson correlation**. **(A)** Reliability (ICC) in terms of the global mean. **(B)** Reliability (ICC) in terms of modularity. The digits “1” through “6” denote the condition of λ being 1, 0.5, 0.1, 0.01, 0.001, and 0.0001 for ASR respectively. The digits “7” and “8' show the average ICC value for ASR over all six conditions and the ICC value of the Pearson correlation respectively.

The resting state fMRI data set of 10 subjects each containing two repeated sessions are used to investigate the reliability of ASR and the Pearson correlation. The values of the ICC index of ASR (under six conditions with different values of λ) and the Pearson correlation are computed in terms of the global mean of the association matrix and the modularity measure, as shown in Figure 10. We do not compute the ICC index in terms of the Silhouette value which involves the AP algorithm. By contrast, the global mean of the association matrix and the modularity measure are directly based on the association matrix, and therefore are more essential in assessing the reliability.

For the global mean of the association matrix, as used in Braun et al. (2012), ASR under all six conditions results in a significantly higher ICC than the Pearson correlation. The ICC value of ASR ranges from 0.569 (λ = 1) to 0.793 (λ = 0.1). The average ICC value over six conditions of ASR reaches 0.676 ± 0.08 while the Pearson correlation yields a relatively lower value of 0.443. For the modularity index, ASR outperforms the Pearson correlation in the cases of λ being 0.5, 0.1, 0.01, and 0.0001. Under the other two conditions with λ being 1 or 0.001, ASR shows lower reliability. The ICC value of ASR ranges from 0.389 (λ = 0.1) to 0.648 (λ = 0.01). The average ICC value over six conditions of ASR reaches 0.383 ± 0.180, which is slightly higher than that of the Pearson correlation with the ICC value being 0.368.

## 4. Discussion

In this paper, we develop a novel scheme to construct the association matrix by ASR instead of the typical Pearson correlation method, and identify connectivity patterns by the AP clustering algorithm. In theory, ASR has two main advantages: (a) ASR is a multivariate method and able to take all nodes into consideration when computing the association, while the Pearson correlation is a bivariate method that can only compute the pairwise association thus ignoring the possible influence from other nodes. (b) The trace LASSO regularizer helps ASR stand out from other existing sparse representation methods, since it can achieve a sparse solution as the ℓ_1_-norm and select correlated nodes as the grouping effect of the ℓ_2_-norm. These advantages make ASR a suitable method to estimate the association matrix of fMRI data. The AP clustering algorithm carries out further analysis by efficiently identifying connectivity patterns based on the obtained global network without setting a threshold to the association matrix. Taken together, the novel scheme provides a new insight into the functional connectivity of human brain.

### 4.1. Performance of estimating global functional connection network

ASR is evaluated on both the simulated and the real fMRI data sets in constructing the association matrix. As illustrated in Figures 3, 4, ASR obtains a substantially sparser solution than the Pearson correlation. That is, unlike the Pearson correlation, most connection strengths obtained by ASR are driven to near zero (e.g., 10^−7^, not exactly zero due to the computational precision). As a result, the essential connections are automatically revealed.

Furthermore, the great sparsity of ASR does not jeopardize its performance of delineating functional connectivity. Indeed, as shown in Figure 3A, ASR successfully captures the underlying network structure of 10 five-node rings. Besides, the *t*-test for the real fMRI data shows that the modularity of ASR is significantly higher than that of the Pearson correlation. Compared with the Pearson correlation, these modules are easier to be interpreted from the perspective of neurophysiology, as shown in Figure 5. This high modularity can be partly due to the grouping effect of ASR, which can select functionally correlated regions altogether.

### 4.2. Comparison of AP clustering results

The traditional strategy to analyze the functional network involves the step of thresholding the association matrix. However, such analysis is heavily dependent on the choice of the threshold value (Zalesky et al., 2010). In this paper, we used the AP clustering algorithm to identify functional network structure without the requirement of applying a threshold. It directly takes the association matrix as input and assigns each node into one cluster.

The quantifiable indexes for both the simulated data (clustering accuracy) and the real fMRI data (Silhouette) clearly indicate that ASR achieves a better clustering quality in terms of accuracy and compactness of clusters. The obtained sub-networks on the real fMRI data suggest that ASR yields a better division of networks that are easier to be interpreted than the Pearson correlation. In a word, the nodes are tightly connected within a cluster while sparsely connected between clusters. The quantifiable index of ASR is significantly higher than the Pearson correlation, although, for visual perception, most networks obtained by the two methods are similar, as illustrated in Figure 7. The choice of the AAL template as the atlas of nodes may partly account for this result, for 90 nodes may not be enough to find subtle difference of details of clusters. Using a more refined atlas or using voxel-wised nodes may find more information.

Results in Figures 7, 8 reveal that RSNs identified by ASR are in accordance with results of some previous studies (van den Heuvel and Pol, 2010, and references therein). Although details within each sub-network are different between subjects due to the inter-subject variability, most subjects report the presence of basal ganglia, DMN, sensorimotor, limbic, frontal-parietal and visual network. The proposed ASR scheme succeeds in identifying RSNs with the AP clustering algorithm, suggesting that ASR is reasonable and feasible to accurately discover functional network structure and underlying connectivity patterns.

### 4.3. Comparison of reliability results

As Figure 10 suggests, ASR achieves a higher ICC value of the global mean than the Pearson correlation under all six different conditions. Note that λ has an explicit impact on the sparsity and thus the ICC value in the reliability analysis. It is interesting that ASR performs better in terms of the ICC value of the global mean than the Pearson correlation regardless of which value of λ is applied. However, the ICC value of modularity tells a slightly different story. ASR achieves a relatively high reliability (ICC = 0.648) only when λ takes 0.01. In other cases, it performs moderately. It may suggest that the reliability of modularity is sensitive to the degree of the sparsity of the association matrix. Nevertheless, the average ICC value of ASR is still slightly higher than that of the Pearson correlation. Generally speaking, ASR is a reliable and stable method to estimate the functional connectivity.

### 4.4. Limitation and future work

Firstly, although ASR exhibits better performance than the Pearson correlation, ASR is a time-consuming method, since hundreds of iterations are usually needed to resolve ASR. Thus, a trade-off between efficiency and accuracy should be considered based on practical problems. Smith et al. (2011) have compared several different approaches to estimating connectivity. Thus, further studies may be undertaken to provide a more comprehensive evaluation of ASR by comparing with more connectivity methods. Secondly, the definition of nodes plays an important role in the delineation of brain networks (Zalesky et al., 2010). In the present work, we only use the AAL template to define nodes, which is a large-scale parcelation and may not be enough to discover subtle differences between subjects. The AAL template may suffer from the low homogeneity of resting state functional signals within each parcel of the AAL template because of the structure-function distinction. The ASR should be further investigated on a voxel-wise level (Zuo et al., 2012) or using nodes defined by some functional parcelation algorithms (Yeo et al., 2011; Betzel et al., 2014). Thirdly, to investigate the connectivity patterns, the AP clustering algorithm is employed following ASR. However, AP, as many other clustering algorithms, can only assign a node to one cluster. Thus, regions that may be involved in multiple networks can only be assigned to one network. Some techniques that take overlapping sub-networks into account (Eavani et al., 2015) may be incorporated with some adjustments to investigate the connectivity patterns in combination with ASR. Finally, We would considering extracting discriminative features based on the connectivity patterns to conduct some classification problems. For example, we could study clinical data and shed new light on the classification of neurologic disorders. In the current experiment we only test our scheme on resting state fMRI data. In the future, we will applying the scheme on some task-related fMRI data.

## 5. Conclusion

In this paper, we develop a novel scheme to estimate brain global functional connection network by using ASR and identify connectivity patterns by the AP clustering algorithm, inspired by recent advances in mathematics and image processing. ASR considers the association between one node and all other nodes simultaneously, where the trace LASSO regularizer ensures the sparsity and grouping effect of the solution controlled by only one parameter. Then the AP clustering algorithm identifies functional sub-networks without the requirement of setting a threshold. Experimental results on both the simulated and the real fMRI data sets show that the proposed scheme is effective and useful to estimate functional connection networks and identify connectivity patterns of human brain. In all, the promising scheme of ASR with AP provide a new insight into investigating the problem of functional connectivity.

### Conflict of interest statement

The authors declare that the research was conducted in the absence of any commercial or financial relationships that could be construed as a potential conflict of interest.
